# Coagulopathy and its effect on treatment and mortality in patients with traumatic intracranial hemorrhage

**DOI:** 10.1007/s00701-021-04808-0

**Published:** 2021-03-23

**Authors:** Janne Kinnunen, Jarno Satopää, Mika Niemelä, Jukka Putaala

**Affiliations:** 1 Department of Neurology, Helsinki University Hospital and University of Helsinki, Haartmaninkatu 4, 00290 Helsinki, Finland; 2 Department of Neurosurgery, Helsinki University Hospital and University of Helsinki, Topeliuksenkatu 5, 00260 Helsinki, Finland

**Keywords:** Anticoagulation, Coagulopathy, Mortality, Outcome, Surgical treatment, Traumatic intracranial hemorrhage

## Abstract

**Background:**

The role of coagulopathy in patients with traumatic brain injury has remained elusive. In the present study, we aim to assess the prevalence of coagulopathy in patients with traumatic intracranial hemorrhage, their clinical features, and the effect of coagulopathy on treatment and mortality.

**Methods:**

An observational, retrospective single-center cohort of consecutive patients with traumatic intracranial hemorrhage treated at Helsinki University Hospital between 01 January and 31 December 2010. We compared clinical and radiological parameters in patients with and without coagulopathy defined as drug- or disease-induced, i.e., antiplatelet or anticoagulant medication at a therapeutic dose, thrombocytopenia (platelet count < 100 E9/L), international normalized ratio > 1.2, or thromboplastin time < 60%. Primary outcome was 30-day all-cause mortality. Logistic regression analysis allowed to assess for factors associated with coagulopathy and mortality.

**Results:**

Of our 505 patients (median age 61 years, 65.5% male), 206 (40.8%) had coagulopathy. Compared to non-coagulopathy patients, coagulopathy patients had larger hemorrhage volumes (mean 140.0 mL vs. 98.4 mL, *p* < 0.001) and higher 30-day mortality (18.9% vs. 9.7%, *p* = 0.003). In multivariable analysis, older age, lower admission Glasgow Coma Scale score, larger hemorrhage volume, and conservative treatment were independently associated with mortality. Surgical treatment was associated with lower mortality in both patients with and without coagulopathy.

**Conclusions:**

Coagulopathy was more frequent in patients with traumatic intracranial hemorrhage presenting larger hemorrhage volumes compared to non-coagulopathy patients but was not independently associated with higher 30-day mortality. Hematoma evacuation, in turn, was associated with lower mortality irrespective of coagulopathy.

**Supplementary Information:**

The online version contains supplementary material available at 10.1007/s00701-021-04808-0.

## Introduction

Traumatic brain injury (TBI) is a major cause of disability and mortality. It has been predicted that TBI will be among the most prevalent cause of mortality and disability worldwide due to increasing incidence and more precise diagnostic protocols [16]. A TBI is classified as either a primary brain injury due to a direct force towards the cranium or as a secondary brain injury resulting from the initial trauma [3]. To prevent and minimize the extent of secondary injury, surgical treatment is warranted in some forms of TBI, depending on the initial type and location of the hemorrhage [11]. Among TBIs, a traumatic intracranial hemorrhage (tICH) (epidural, subdural, subarachnoidal, or parenchymal) appears in almost 50% of cases [22].

The state of coagulopathy is a factor that can cause more severe intracranial bleeding and further increase mortality and disability [7]. Coagulopathy can be related to an underlying disease or to the use of antithrombotic medication, i.e., antiplatelets or anticoagulants. Coagulopathy can be defined based on cut-off values of international normalized ratio (INR) > 1.2, platelet count (PLT) < 100 E9/L, or thromboplastin time (TT) < 60% [2, 38].

We aimed to study the prevalence of coagulopathy in patients with tICH, clinical features of these patients, and how coagulopathy affects their treatment and mortality.

## Methods

We performed a retrospective, observational, single-center cohort study of consecutive patients with tICH treated at the Helsinki University Hospital between 01 January and 31 December 2010. Our hospital serves as the only neurosurgical unit with a 24/7 service for operative and neurocritical care for a defined catchment population of 2.0 million. Ethics Committee of the Helsinki and Uusimaa Hospital District approved the study and relevant institutional permission was obtained. Patient consents were waived since the study is based on data gathered for routine care without further patient contacts.

**Fig. 1:**
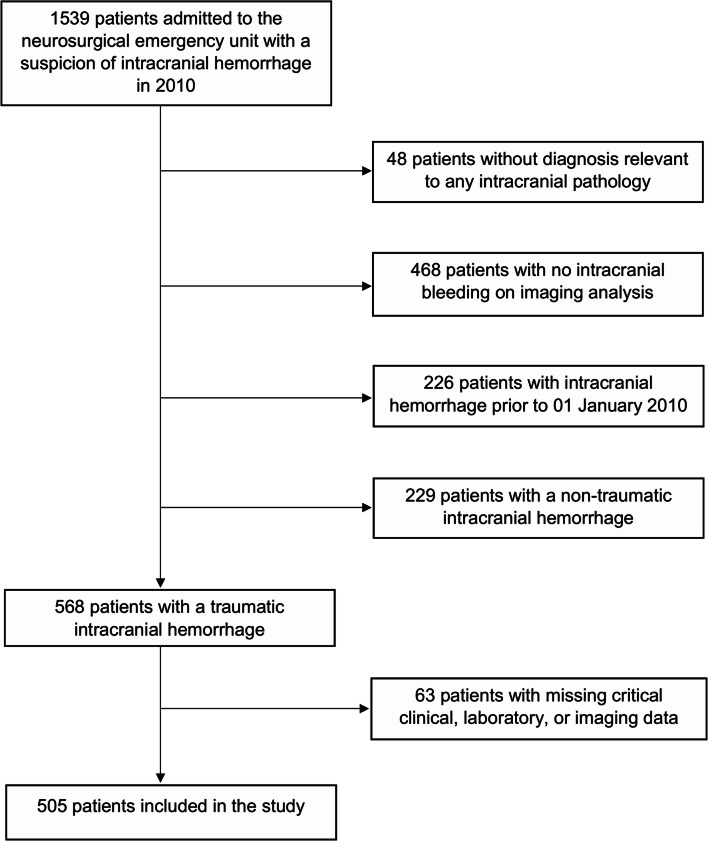
Patient selection flowchart

We screened all patients with a suspected tICH from the hospital discharge registry, using International Classification of Diseases, 10th revision (ICD-10) codes S06.*, I60.*, I61.*, and I62.*. After screening, we subsequently excluded patients with no evidence of intracranial hemorrhage, non-traumatic intracranial hemorrhage, or insufficient clinical or radiological data to make judgment (Fig. 1).

**Fig. 2:**
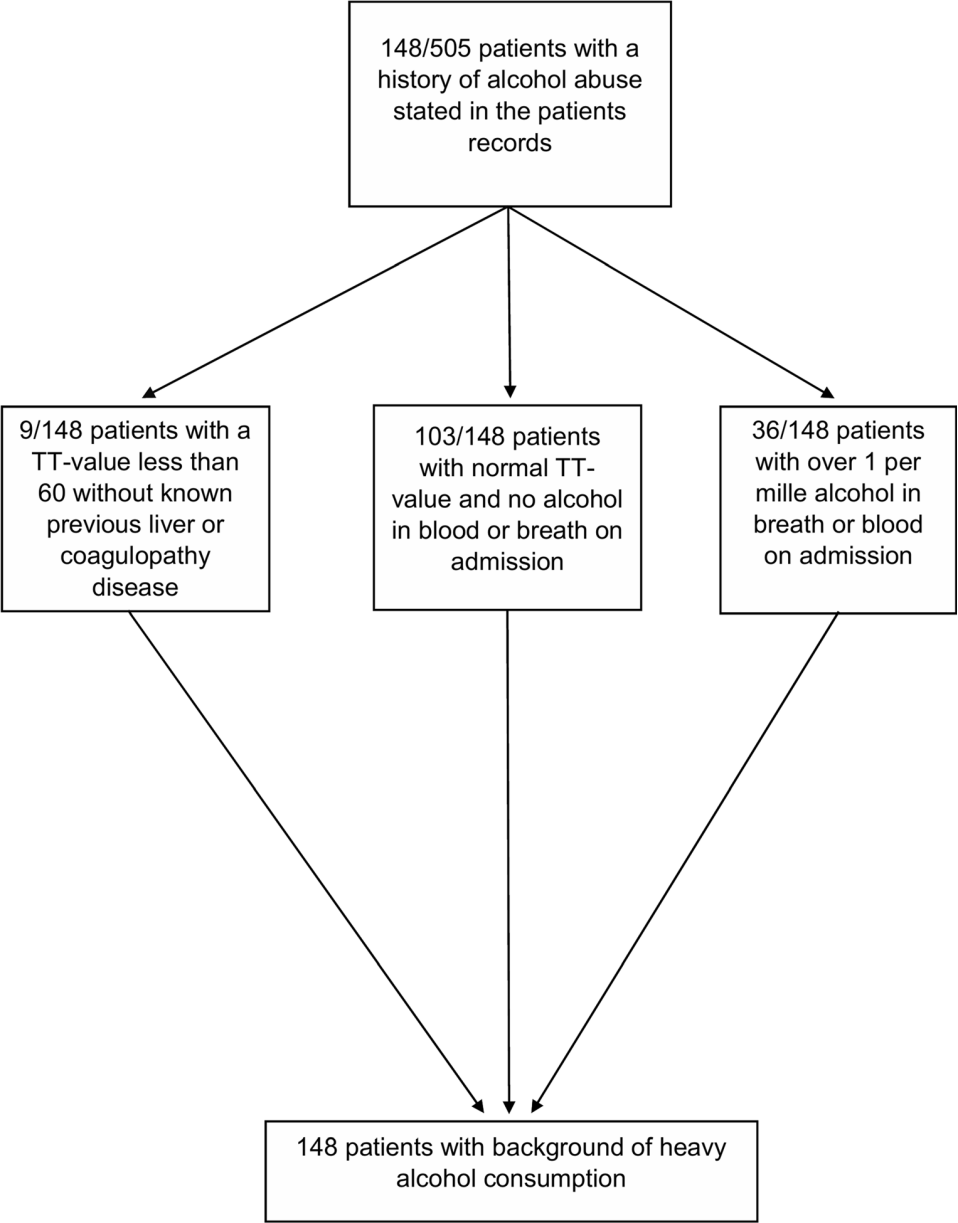
Patient screening for alcohol abuse

Data was collected from electronic patient charts, and laboratory and imaging archives. Comorbidities considered were hypertension, coronary heart disease, and atrial fibrillation (patient history or electrocardiogram measurement during the index hospital admission). Use of antiplatelet or anticoagulation medication prior to hospitalization was recorded. Of routine laboratory results, we recorded PLT, INR, and TT for this study. We estimated the use of alcohol based on patient records and described related admission findings for these patients (Fig. 2). Finland has good and reliable register-based data on alcohol-related hospitalization, which has been stated in alcohol consumption related articles before [21]. Therefore, notes of abundant use of alcohol in patient records come from well-documented hospital or other healthcare visits. Further, the current history of alcohol use was described on their admission charts, which all were manually evaluated for this study by an investigator (J.K.). WHO defines average volume drinking categories by consumption of pure alcohol per sex, in which daily amounts range from 0 to 40 g for women and 0 to 60 g for men [8]. According to Finnish national guidelines, one standard alcohol drink is defined as 12 g, and > 20 g/day or 12 to 16 drinks/week and > 40 g/day or 23 to 24 drinks/week for women and men, respectively, is defined as heavy drinking [14, 31].

Upon hospital arrival, all patients were assessed with Glasgow Coma Scale (GCS) score and they underwent cranial imaging by computerized tomography (CT). CT images were reviewed by a staff neuroradiologist, who identified the intracranial hemorrhage. The decision of surgical treatment was made by a neurosurgeon on duty. Hematoma evacuation was considered as a neurosurgical operation. There were only few ventriculostomies, which we registered as an additional procedure. We also recorded the use of any medicinal method aiming to correct coagulopathy, including fresh frozen plasma, specific coagulation factor concentrate, and red blood cell infusion.

All CT images were re-evaluated by an investigator (J.K.) to confirm intracranial hemorrhage. In case of multiple hemorrhagic components, the main component was chosen based on clinical grounds, and its volume was measured and calculated using ABC/2 and XYZ/2 methods [18, 33].

We defined coagulopathy by the use of antiplatelet or anticoagulant medication at a therapeutic dose or by admission laboratory cut-off values PLT < 100 E9/L, INR > 1.2, or TT < 60%. In Finnish laboratories, TT value is defined as a thromboplastin time and the result is expressed as a percentage of coagulation activity, where 100% is determined by a reference plasma sample with full coagulation activity. An individual plasma sample is compared to that forming a ratio, where 70 to 130% is a reference range for normal level of coagulation activity. Subgroups of coagulopathy were further categorized as (1) no coagulopathy, (2) medication-induced by use of antithrombotic medication, (3) spontaneous by known disease or without any specific causation, and (4) coagulopathy by both medication and laboratory findings.

We used 30-day all-cause mortality as the primary outcome. Mortality data came from the Statistics Finland.

For statistical analysis, we used SPSS 22.0 (IBM Corp., Armonk, NY, USA). Normality of distributions was assessed. We used Student’s *t* test for normally distributed continuous variables and Mann-Whitney *U* test for non-normally distributed continuous variables. Pearson’s chi-square and Fisher’s exact test allowed comparison of the categorical variables. Multivariable logistic regression was used to assess factors associated with coagulopathy and 30-day all-cause mortality. Covariable selection was based on existing literature, including demographics (age, gender), main cardiovascular comorbidities (hypertension, coronary heart disease, atrial fibrillation), clinical features (GCS, hemorrhage volume), and neurosurgical intervention. We also performed a sensitivity analysis by removing treatment modalities from the multivariable analysis. A two-sided *p* < 0.05 was considered significant. Subgroup analyses were performed for the different groups of coagulopathy states as categorized above, and separately for patients with coagulopathy where they were considered as one group regardless of the etiology of coagulopathy. Additionally, we did an analysis, in which we defined the patients’ current use of alcohol being either alcohol abuse or not.

## Results

A total of 1539 patients were admitted to the neurosurgical emergency unit with a suspicion of intracranial hemorrhage in 2010. After exclusions, 505 patients with imaging-verified tICH (epidural, subdural, subarachnoidal, or parenchymal) were included in the study (Fig. 1). Of the included patients, 361 (65.9%) were male, median age was 61 years, 272 (53.9%) underwent neurosurgical hematoma evacuation, and 135 (26.7%) were transferred from another hospital to the neurosurgical unit. On admission phase, delay from the index traumatic event and symptom onset to hospitalization was 0 (IQR 0–1) days, which was found statistically significant on mortality (*p* = 0.002). Delays from symptom onset and hospital admission to hematoma evacuation were 1 (IQR 0–7) and 0 (IQR 0–1) days, respectively. Of them, the previous one was statistically significant (*p* = 0.002) in relation to mortality within the patient group of neurosurgical operation.

Coagulopathy was observed in 206 patients (40.1%). We did not find any patients with previously known hereditary coagulopathies. The majority of coagulopathy patients were male (66.5%), and over a half (60.2%) underwent surgical hematoma operation. In comparison to non-coagulopathy patients, those with coagulopathy were older (69.0 vs. 57.5 years), had more comorbidities such as hypertension or atrial fibrillation or coronary heart disease, and had larger hemorrhage volumes. In multivariable analysis, age groups from 65 years above, atrial fibrillation, coronary heart disease, and alcohol abuse were associated with presence of coagulopathy (Table 1).

**Table 1:** Comparison of clinical and neuroimaging characteristics between patients with and without coagulopathy. Multivariable odds ratios for mortality from a logistic regression model adjusted for the given variables

Variable	Coagulopathy *N* = 206 (40.8%)	No coagulopathy *N* = 299 (59.2%)	*p*	Multivariable odds ratio (95% confidence interval)	Multivariable *p*
Male gender	137 (66.5%)	194 (64.9%)	0.775	1.131 (0.700–1.825)	0.615
Age, mean (95% CI)	69.0 (66.8–71.3)	57.5 (55.0–60.0)	< 0.001	NA^a^	NA^a^
Age group			< 0.001		
< 50	27 (13.1%)	113 (37.8%)		Reference	
50–64	47 (22.8%)	100 (33.4%)		1.194 (0.643–2.218)	0.575
65–79	82 (39.8%)	53 (17.7%)		3.862 (1.967–7.582)	< 0.001
≥ 80	50 (24.3%)	33 (11.0%)		3.055 (1.349–6.919)	0.007
Admission GCS			0.448		
13–15	130 (63.1%)	180 (60.2%)		Reference	
9–12	26 (12.6%)	32 (10.7%)		1.135 (0.555–2.323)	0.728
3–8	50 (24.3%)	87 (29.1%)		1.216 (0.727–2.032)	0.457
Hypertension	90 (43.7%)	73 (24.4%)	< 0.001	1.214 (0.737–2.001)	0.446
Atrial fibrillation	64 (31.1%)	6 (2.0%)	< 0.001	14.416 (5.794–35.873)	< 0.001
Coronary heart disease	53 (25.7%)	10 (3.3%)	< 0.001	6.312 (2.891–13.785)	< 0.001
Alcohol abuse	63 (30.6%)	85 (28.4%)	0.620	2.150 (1.280–3.611)	0.004
Antiplatelet medication	99 (48.1%)	NA^b^	NA^b^	NA^b^	NA^b^
Anticoagulation medication	73 (35.4%)	NA^b^	NA^b^	NA^b^	NA^b^
Thrombocyte level (100 × 10^9^/L), mean (95% CI)	202 (182–222)	NA^b^	NA^b^	NA^b^	NA^b^
INR value, mean (95% CI)	2.4 (2.1–2.7)	NA^b^	NA^b^	NA^b^	NA^b^
Thromboplastin time (%), mean (95% CI)	41 (35–48)	NA^b^	NA^b^	NA^b^	NA^b^
Hematoma evacuation	124 (60.2%)	148 (49.5%)	0.011	0.949 (0.540–1.671)	0.857
Ventriculostomy	6 (2.9%)	8 (2.7%)	0.540	2.115 (0.594–7.526)	0.247
Hemorrhage volume (mL), mean (95% CI)	140.0 (125.4–154.4)	98.4 (86.4–110.4)	< 0.001	NA^a^	NA^a^
Hemorrhage volume (mL)			< 0.001		
0–50	69 (33.5%)	151 (50.5%)		Reference	
51–100	23 (11.2%)	45 (15.1%)		0.706 (0.337–1.478)	0.356
101–200	67 (32.5%)	60 (20.1%)		1.328 (0.675–2.613)	0.411
> 200	47 (22.8%)	43 (14.4%)		1.092 (0.514–2.317)	0.819

*p*, *p*-value; *CI*, confidence interval; *GCS*, Glasgow Coma Scale. NA^a^ = not included in the regression model due to categorized parameter of the same value, NA^b^ = not analyzed due to medication itself being a defining factor for coagulopathy

Of all 505 patients included in the study, a total of 68 patients (13.5%) died within 30 days. In univariable analysis, those who died were older, had lower GCS scores, and larger hemorrhage volumes, but they had undergone less often hematoma evacuation. In multivariable analysis, the same variables remained associated with 30-day mortality. The presence of coagulopathy did not differ between those who survived and died within 30 days (Table 2). In the sensitivity analysis, differentiated coagulopathy etiologies did not show any statistical significance in relation to 30-day mortality. Also, a neurosurgical hematoma evacuation and coagulopathy correction were excluded together and separately, and no association between coagulopathy and 30-day mortality was observed (see Tables, Online Resource 1–4, analysis for entire study cohort).

**Table 2:** Univariable and multivariable analysis of factors associated with 30-day mortality in the entire study cohort (*n* = 505). Odds ratios from a logistic regression model: analyzing each variable separately and adjusted for all the given variables

Variable	Alive *N* = 437 (86.5%)	Dead *N* = 68 (13.5%)	Univariable OR (95% CI)	Univariable *p*	Multivariable OR (95% CI)	Multivariable *p*
Male gender	282 (64.5%)	49 (72.1%)	1.418 (0.806–2.493)	0.226	1.550 (0.750–3.202)	0.237
Age, mean (95% CI)	62.3 (60.4–64.3)	63.5 (58.8–68.2)	1.015 (1.001–1.029)	0.035	NA^a^	NA^a^
Age group						
< 50	128 (29.3%)	12 (17.6%)	Reference		Reference	
50–64	128 (29.3%)	19 (27.9%)	1.583 (0.738–3.396)	0.238	1.452 (0.565–4)	0.438
65–79	114 (26.1%)	21 (30.9%)	1.965 (0.926–4.172)	0.079	3.405 (1.197–9.684)	0.022
≥ 80	67 (15.3%)	16 (23.5%)	2.547 (1.139–5.696)	0.023	5.881 (1.740–19.870)	0.004
Admission GCS						
13–15	294 (67.3%)	16 (23.5%)	Reference		Reference	
9–12	51 (11.7%)	7 (10.3%)	2.522 (0.989–6.435)	0.053	2.634 (0.901–7.703)	0.077
3–8	92 (21.1%)	45 (66.2%)	8.988 (4.851–16.652)	< 0.001	15.351 (6.894–34.185)	< 0.001
Hypertension	142 (32.5%)	21 (30.9%)	0.928 (0.534–1.612)	0.791	0.793 (0.370–1.697)	0.550
Atrial fibrillation	55 (12.6%)	15 (22.1%)	1.966 (1.037–3.725)	0.038	1.653 (0.638–4.296)	0.300
Coronary heart disease	49 (11.2%)	14 (20.6%)	2.053 (1.062–3.967)	0.032	2.004 (0.792–5.072)	0.142
Alcohol abuse	122 (27.9%)	26 (38.2%)	1.598 (0.939–2.721)	0.084	1.964 (0.917–4.205)	0.082
Coagulopathy	167 (38.2%)	39 (57.4%)	2.174 (1.295–3.649)	0.003	1.572 (0.729–3.391)	0.249
Thrombocyte level (100 × 10^9^/L), mean (95% CI)	210 (192–228)	186 (153–220)	0.993 (.989–0.997)	< 0.001	NA^a^	NA^a^
INR value, mean (95% CI)	1.9 (1.7–2.1)	2.4 (1.6–3.3)	1.249 (0.928–1.680)	0.142	NA^a^	NA^a^
Thromboplastin time (%), mean (95% CI)	60 (52–69)	49 (31–67)	0.985 (0.975–0.994)	0.001	NA^a^	NA^a^
Coagulopathy correction	152 (34.8%)	30 (44.1%)	1.480 (0.882–2.484)	0.137	0.725 (0.340–1.545)	0.404
Hematoma evacuation	248 (56.8%)	24 (35.3%)	0.416 (0.244–0.708)	0.001	0.138 (0.061–0.316)	< 0.001
Ventriculostomy	11 (2.5%)	3 (4.4%)	1.787 (0.486–6.578)	0.382	2.824 (0.626–12.737)	0.177
Hemorrhage volume (mL), mean (95% CI)	111.9 (102.0–121.8)	142.0 (113.2–170.7)	1.004 (1.001–1.006)	0.002	NA^a^	NA^a^
Hemorrhage volume (mL)						
0–50	201 (46.0%)	19 (27.9%)	Reference		Reference	
51–100	55 (12.6%)	13 (19.1%)	2.500 (1.162–5.378)	0.019	2.689 (1.027–7.039)	0.044
101–200	109 (24.9%)	18 (26.5%)	1.747 (0.880–3.467)	0.111	3.807 (1.415–10.241)	0.008
> 200	72 (16.5%)	18 (26.5%)	2.645 (1.315–5.318)	0.006	4.477 (1.562–12.836)	0.005

*OR*, odds ratio; *p*, *p*-value; *CI*, confidence interval; *GCS*, Glasgow Coma Scale. NA^a^ = not included in the regression model due to categorized parameter of the same value

When coagulopathy was divided into subgroups, a univariable analysis showed higher 30-day mortality for those with both medication-induced and spontaneous coagulopathy (see Table, Online Resource 5, analysis for coagulopathy subgroups). However, in multivariable analysis, this difference was not significant (see Table, Online Resource 6, analysis for entire study cohort with coagulopathy subgroups), and not even in the sensitivity analysis with and without adjustment for neurosurgical hematoma evacuation or coagulopathy correction (see Tables, Online Resource 7–9, analysis for entire study cohort with coagulopathy subgroups).

Of the 206 patients with coagulopathy, 39 (18.9%) patients died within 30 days. In univariable analysis, patients who died had lower admission GCS score, were less often operated for hematoma evacuation, but underwent more frequently ventriculostomy. In multivariable analysis, age ≥ 80 years, admission GCS score between 3 and 8, as well as ventriculostomy were associated with higher 30-day mortality, whereas hematoma evacuation was the only factor associated with a lower 30-day mortality (Table 3).

**Table 3:** Univariable and multivariable analysis of factors associated with 30-day mortality in the coagulopathy subcohort (*n* = 206). Odds ratios from a logistic regression model: analyzing each variable separately and adjusted for all the given variables

Variable	Alive *N* = 167 (81.1%)	Dead *N* = 39 (18.9%)	Univariable OR (95% CI)	Univariable *p*	Multivariable OR (95% CI)	Multivariable *p*
Male gender	109 (65.3%)	28 (71.8%)	1.354 (0.629–2.916)	0.438	1.367 (0.519–3.602)	0.527
Age, mean (95% CI)	66.9 (64.4–69.4)	68.3 (62.2–74.5)	1.005 (0.984–1.027)	0.629	NA^a^	NA^a^
Age group						
< 50	24 (14.4%)	3 (7.7%)	Reference		Reference	
50–64	37 (22.2%)	10 (25.6%)	2.162 (0.539–8.670)	0.276	2.791 (0.486–16.016)	0.250
65–79	68 (40.7%)	14 (35.9%)	1.647 (0.435–6.234)	0.462	4.459 (0.756–26.316)	0.099
≥ 80	38 (22.8%)	12 (30.8%)	2.526 (0.646–9.887)	0.183	8.492 (1.189–60.627)	0.033
Admission GCS						
13–15	116 (69.5%)	14 (35.9%)	Reference		Reference	
9–12	20 (12.0%)	6 (15.4%)	2.486 (0.855–7.230)	0.095	2.342 (0.690–7.947)	0.172
3–8	31 (18.6%)	19 (48.7%)	5.078 (2.291–11.257)	< 0.001	6.476 (2.291–18.302)	< 0.001
Hypertension	74 (44.3%)	16 (41.0%)	0.874 (0.431–1.774)	0.710	0.633 (0.259–1.548)	0.316
Atrial fibrillation	49 (29.3%)	15 (38.5%)	1.505 (0.728–3.111)	0.270	1.536 (0.574–4.112)	0.393
Coronary heart disease	39 (23.4%)	14 (35.9%)	1.838 (0.872–3.876)	0.110	2.492 (0.972–6.393)	0.057
Alcohol abuse	49 (29.3%)	14 (35.9%)	1.349 (0.647–2.810)	0.425	2.370 (0.797–7.049)	0.121
Thrombocyte level (100 × 10^9^/L), mean (95% CI)	208 (184–231)	184 (144–224)	NA^b^	NA^b^	NA^b^	NA^b^
INR value, mean (95% CI)	2.3 (2.0–2.5)	2.8 (1.7–3.8)	NA^b^	NA^b^	NA^b^	NA^b^
Thromboplastin time (%), mean (95% CI)	43 (35–50)	37 (21–52)	NA^b^	NA^b^	NA^b^	NA^b^
Coagulopathy correction	91 (54.5%)	24 (61.5%)	1.336 (0.655–2.727)	0.426	1.166 (0.453–3.001)	0.750
Hematoma evacuation	108 (64.7%)	16 (41.0%)	0.380 (0.186–0.775)	0.008	0.214 (0.068–0.675)	0.008
Ventriculostomy	3 (1.8%)	3 (7.7%)	4.556 (0.883–23.495)	0.070	11.941 (1.602–89.020)	0.016
Hemorrhage volume (mL), mean (95% CI)	119.4 (104.5–134.4)	127.8 (91.8–163.7)	1.001 (0.997–1.004)	0.639	NA^a^	NA^a^
Hemorrhage volume (mL)						
0–50	57 (34.1%)	12 (30.8%)	Reference		Reference	
51–100	17 (10.2%)	6 (15.4%)	1.676 (0.547–5.137)	0.366	0.643 (0.167–2.470)	0.520
101–200	56 (33.5%)	11 (28.2%)	0.933 (0.380–2.289)	0.880	1.342 (0.343–5.246)	0.672
> 200	37 (22.2%)	10 (25.6%)	1.284 (0.504–3.272)	0.601	1.482 (0.367–5.982)	0.432

*OR*, odds ratio; *p*, *p*-value; *CI*, confidence interval; *GCS*, Glasgow Coma Scale. NA^a^ = not included in the regression model due to categorized parameter of the same value, NA^b^ = not analyzed due to medication itself being defining factor for the coagulopathy

In the secondary analysis based on alcohol use status, we divided the patients with alcohol abuse to subgroups for univariable analysis of 30-day mortality. Only one group, in which patients had a history of previous heavy use of alcohol and their admission laboratory values indicated coagulopathy, showed higher 30-day mortality (see Table, Online Resource 10, analysis for alcohol abuse subgroups). We also included the same subgroups in a multivariable analysis, in which older age groups, lower GCS score, and higher hemorrhage volumes were independently associated with higher 30-day mortality. In the same analysis, hematoma evacuation remained associated with a lower 30-day mortality (see Table, Online Resource 11, analysis for entire study cohort with alcohol abuse subgroups).

## Discussion

Our study showed that coagulopathy was frequent in patients with tICH, but it did not convey independent effect on 30-day mortality. Interestingly, larger hemorrhage volumes were not associated with coagulopathy after adjustment for confounders, albeit they indicated higher mortality. Neurosurgical hematoma evacuation was associated with a lower 30-day mortality, irrespective of coagulopathy, and adjusted for known factors associated with tICH mortality.

### Prevalence of coagulopathy in tICH

In our study, we used basic concepts to define coagulopathy by routine laboratory values, being valid for detecting coagulopathy state [38]. We also combined laboratory values and use of antithrombotic medication to catch a wider population of coagulopathy. However, there are more advanced methods of measuring coagulopathy, which have the advantage over more traditional methods of measuring coagulation potential by laboratory values [5, 28, 32].

The prevalence of coagulopathy in our study was approximately 40%. In other studies, the proportion of coagulopathy defined with more advanced methods was as high as 75%, also reporting that the discrepancy between conventional and advanced methods ranged from 25 to 45% [10, 34]. Those figures correspond fairly well with our findings.

Coagulopathy is associated with progressive hemorrhage and higher mortality in patients with TBI [20]. However, in our study, there was no association between coagulopathy and larger hemorrhage volume. Regarding the association between coagulopathy and 30-day mortality, we categorized the groups of coagulopathy to those of (1) no coagulopathy, (2) medication-induced by use of antithrombotic medication, (3) spontaneous by known disease or without any specific causation, and (4) coagulopathy by both medication and laboratory findings. Of these, the latter group (4) was the only one with statistically significant association with mortality in the coagulopathy subgroup analysis. However, in multivariable analysis, coagulopathy subtypes were not independently associated with higher mortality, which is in conjunction with our main findings that coagulopathy does not associate independently to higher mortality in patients with tICH.

### Mortality in tICH related to coagulopathy

Previous studies show tendency that tICH carry 40 to 50% mortality from all-cause trauma-based fatal events [17, 24, 27]. In more targeted cohorts of tICH patients, the fatality rate for tICH events in general was 15%, and it ranged from 10 for non-coagulopathy group to nearly 50% in a coagulopathy group [15, 24]. Our study resembles a similar scale with the values of overall mortality being approximately 14% for tICH events and with non-coagulopathy group mortality being close to 10%, but it is discrepant with the coagulopathy fatality rate when ours showed 20% mortality.

In contrast to our findings, a recent study has found statistical significance between coagulopathy and mortality in tICH patient cohort, yet, the mortality was defined to be in-hospital occurrence [24]. To our slight surprise, the volume of intracranial hemorrhage had no association with mortality in coagulopathy group analysis. Nevertheless, in our main analysis, it did have association with higher mortality. One explanation for these observations might be that patients with coagulopathy are more intensively monitored and treated due to their expectedly higher risk of neurological and medical complications, and death [39].

These variations in general could be due to different definitions of coagulopathy. Also, the treatment methods for coagulopathy have improved during the last decades with more efficient ways of point-of-care testing of coagulopathy and more goal-directed coagulation management by different pro-coagulant products [10, 29]. In our study, we did not differentiate between coagulopathy correction methods such as prothrombin complexes or different coagulation factor products as we made it a dichotomous variable. Subsequently, we did not find any association between coagulopathy and mortality, even after detailed sensitivity analyses. Moreover, our study suggests that hematoma evacuation was associated with lower mortality in a broad range of patients with coagulopathy.

### Clinical features

Some studies suggest that alcohol has a protective effect in TBIs [1, 26]. The role of alcohol in coagulopathy has been shown to be a risk factor in chronic alcohol use and in acute emergency setting [6, 25, 37]. Our study is in accordance with these studies, as we did not find association between alcohol abuse and mortality in multivariable analysis. However, heavy drinking was associated with coagulopathy.

In our study, older age was associated with higher 30-day mortality in the entire cohort, but in the coagulopathy group, there was no association with age and mortality. Among elderly, there is a higher risk of tICH, in particular with the use of antithrombotic medication, and the outcomes are poorer within this age group [30].

Lower GCS score is associated with higher mortality in patients with tICH [23]. Our study supports this evidence. Also, hypertension has been found to associate with tICH [13]. In our study, hypertension was the most prevalent comorbidity, yet hypertension was not associated with higher 30-day mortality. Surgical treatment indications vary between the types of intracranial hemorrhages [4, 9]. Furthermore, there is dispute regarding the optimal surgical method related to intracranial hemorrhage types and benefit of each surgical method [35]. Also, the effectiveness of surgical approach is highly dependent on the selected study and no clear consensus is available [35]. Variation in outcomes of coagulopathy patients seems to be multifactorial and not as clear as it has been indicated in previous patient cohort studies [12]. Based on our findings, it is not possible to make conclusions regarding the choice of surgical treatment or their separate effect on the outcome since we pooled various forms of tICH. Moreover, we did not differentiate between surgical methods but instead used a binary variable for patients having surgical hematoma evacuation or not. There are also some patients with chronic subdural hemorrhages in the patient population treated by a trepanation, known to carry less risk for complications [19, 36].

### Strengths and limitations

In our study, the main limitation is its retrospective and single-center nature. However, a notable major strength is that we could include consecutive patients as our study is entirely free of consent bias. Many of our patients with head trauma may have harbored comorbidities and other factors, such as alcoholism, leading to the index traumatic event and hospitalization, which may have hampered obtaining a consent. However, there still might exist some selection bias due to fact that the patient cohort is from a tertiary university facility, which sorts out the milder cases of head trauma. We excluded patients without CT positive intracranial hemorrhage; therefore, no further data were available for these patients. Furthermore, majority of our patients with coagulopathy due to medication were using warfarin for atrial fibrillation, while currently, the use of oral anticoagulation with non-vitamin K antagonist anticoagulants is preferred over warfarin. Also, our cohort represents a decade old practice in treatment protocols of the patient group in question. This choice was made due to methodological reasons in pursuing adequate follow-up period, as we will later perform further analysis concentrating on post-trauma rehabilitation and health economic issues. Yet, our cohort will serve as a reference point when comparing newer knowledge and advanced treatment options for this patient group.

## Conclusion

Coagulopathy was a frequent feature in patients with tICH and larger hemorrhage volumes. However, coagulopathy per se conveyed no higher 30-day mortality in these patients. Surgical hematoma evacuation was associated with lower mortality in patients with or without coagulopathy.

## Supplementary Information

ESM 1 (DOCX 15 kb)

ESM 2 (DOCX 13 kb)

ESM 3 (DOCX 13 kb)

ESM 4 (DOCX 13 kb)

ESM 5 (DOCX 12 kb)

ESM 6 (DOCX 14 kb)

ESM 7 (DOCX 14 kb)

ESM 8 (DOCX 14 kb)

ESM 9 (DOCX 14 kb)

ESM 10 (DOCX 12 kb)

ESM 11 (DOCX 14 kb)
