# Supplementary material for: Coagulopathy and its effect on treatment and mortality in patients with traumatic intracranial hemorrhage
Source: Acta Neurochir (Wien). 2021 Mar 23;163(5):1391–401. doi: 10.1007/s00701-021-04808-0 (PMC8053656; doi:10.1007/s00701-021-04808-0)
Supplement: Supplementary file 1 — (DOCX 15 kb) [file 701_2021_4808_MOESM1_ESM.docx]

**Online Resource 1. Table.**

Univariable and multivariable analysis of factors associated with 30-day mortality in the entire study cohort (n=505). Sensitivity analysis with indicating coagulopathy etiologies. Odds ratios from a logistic regression model: adjusted for all the given variables.

| **Variable** | **Alive**  **N=437 (86.5%)** | **Dead**  **N=68 (13.5%)** | **Univariable OR (95% CI)** | **Univariable p** | **Multivariable OR (95% CI)** | **Multivariable p** |
| --- | --- | --- | --- | --- | --- | --- |
| Male gender | 282 (64.5%) | 49 (72.1%) | 1.418 (0.806-2.493) | 0.226 | 0.638 (0.307-1.326) | 0.228 |
| Age, mean (95% CI) | 62.3 (60.4-64.3) | 63.5 (58.8-68.2) | 1.015 (1.001-1.029) | 0.035 | NA^a^ | NA^a^ |
| Age group |  |  |  |  |  |  |
| <50 | 128 (29.3%) | 12 (17.6%) | Reference |  | Reference |  |
| 50-64 | 128 (29.3%) | 19 (27.9%) | 1.583 (0.738-3.396) | 0.238 | 1.461 (0.568-3.759) | 0.432 |
| 65-79 | 114 (26.1%) | 21 (30.9%) | 1.965 (0.926-4.172) | 0.079 | 3.393 (1.181-9.749) | 0.022 |
| ≥80 | 67 (15.3%) | 16 (23.5%) | 2.547 (1.139-5.696) | 0.023 | 5.879 (1.722-20.064) | 0.005 |
| Admission GCS |  |  |  |  |  |  |
| 13-15 | 294 (67.3%) | 16 (23.5%) | Reference |  | Reference |  |
| 9-12 | 51 (11.7%) | 7 (10.3%) | 2.522 (0.989-6.435) | 0.053 | 2.498 (0.845-7.384) | 0.023 |
| 3-8 | 92 (21.1%) | 45 (66.2%) | 8.988 (4.851-16.652) | <0.001 | 15.203 (6.839-33.799) | <0.001 |
| Hypertension | 142 (32.5%) | 21 (30.9%) | 0.928 (0.534-1.612) | 0.791 | 1.240 (0.575-2.672) | 0.583 |
| Atrial fibrillation | 55 (12.6%) | 15 (22.1%) | 1.966 (1.037-3.725) | 0.038 | 0.644 (0.176-2.354) | 0.506 |
| Coronary heart disease | 49 (11.2%) | 14 (20.6%) | 2.053 (1.062-3.967) | 0.032 | 0.521 (0.198-1.373) | 0.187 |
| Alcohol abuse | 122 (27.9%) | 26 (38.2%) | 1.598 (0.939-2.721) | 0.084 | 0.498 (0.233-1.066) | 0.073 |
| Coagulopathy |  |  |  |  |  |  |
| Anticoagulation medication | 61 (14.0%) | 14 (20.6%) | 1.598 (0.837-3.052) | 0.156 | 0.931 (0.214-4.049) | 0.924 |
| Antiplatelet medication | 79 (18.1%) | 20 (29.4%) | 1.888 (1.062-3.358) | 0.030 | 0.690 (0.289-1.648) | 0.404 |
| Coagulopathy by laboratory values | 100 (22.9%) | 25 (36.8%) | 2.000 (1.162-3.443) | 0.012 | 0.630 (0.259-1.534) | 0.309 |
| Thrombocyte level (100 x 10^9^/l), mean (95% CI) | 210 (192-228) | 186 (153-220) | 0.993 (.989-0.997) | <0.001 | NA^a^ | NA^a^ |
| INR value, mean (95% CI) | 1.9 (1.7-2.1) | 2.4 (1.6-3.3) | 1.249 (0.928-1.680) | 0.142 | NA^a^ | NA^a^ |
| Thromboplastin time (%), mean (95% CI) | 60 (52-69) | 49 (31-67) | 0.985 (0.975-0.994) | 0.001 | NA^a^ | NA^a^ |
| Coagulopathy correction | 152 (34.8%) | 30 (44.1%) | 1.480 (0.882-2.484) | 0.137 | 0.686 (0.305-1.543) | 0.362 |
| Hematoma evacuation | 248 (56.8%) | 24 (35.3%) | 0.416 (0.244-0.708) | 0.001 | 0.143 (0.062-0.327) | <0.001 |
| Ventriculostomy | 11 (2.5%) | 3 (4.4%) | 1.787 (0.486-6.578) | 0.382 | 2.956 (0.645-13.554) | 0.163 |
| Hemorrhage volume (ml), mean (95% CI) | 111.9 (102.0-121.8) | 142.0 (113.2-170.7) | 1.004 (1.001-1.006) | 0.002 | NA^a^ | NA^a^ |
| Hemorrhage volume (ml) |  |  |  |  |  |  |
| 0-50 | 201 (46.0%) | 19 (27.9%) | Reference |  | Reference |  |
| 51-100 | 55 (12.6%) | 13 (19.1%) | 2.500 (1.162-5.378) | 0.019 | 2.682 (0.998-6.922) | 0.051 |
| 101-200 | 109 (24.9%) | 18 (26.5%) | 1.747 (0.880-3.467) | 0.111 | 3.697 (1.362-10.035) | 0.010 |
| >200 | 72 (16.5%) | 18 (26.5%) | 2.645 (1.315-5.318) | 0.006 | 4.270 (1.484-12.290) | 0.007 |

OR = odds ratio, p = p-value, CI = confidence interval, GCS = Glasgow Coma Scale, NA^a^ = not included in the regression model due to categorized parameter of the same value
