# Supplementary material for: Coagulopathy and its effect on treatment and mortality in patients with traumatic intracranial hemorrhage
Source: Acta Neurochir (Wien). 2021 Mar 23;163(5):1391–401. doi: 10.1007/s00701-021-04808-0 (PMC8053656; doi:10.1007/s00701-021-04808-0)
Supplement: Supplementary file 3 — (DOCX 13 kb) [file 701_2021_4808_MOESM3_ESM.docx]

**Online Resource 3. Table.**

Multivariable analysis of factors associated with 30-day mortality in the entire study cohort (n=505). Sensitivity analysis with coagulopathy correction, without neurosurgical hematoma evacuation. Odds ratios from a logistic regression model: adjusted for all the given variables.

| **Variable** | **Alive**  **N=437 (86.5%)** | **Dead**  **N=68 (13.5%)** | **Multivariable OR (95% CI)** | **Multivariable p** |
| --- | --- | --- | --- | --- |
| Male gender | 282 (64.5%) | 49 (72.1%) | 1.424 (0.715-2.837) | 0.314 |
| Age, mean (95% CI) | 62.3 (60.4-64.3) | 63.5 (58.8-68.2) | NA^a^ | NA^a^ |
| Age group |  |  |  |  |
| <50 | 128 (29.3%) | 12 (17.6%) | Reference |  |
| 50-64 | 128 (29.3%) | 19 (27.9%) | 1.568 (0.625-3.933) | 0.338 |
| 65-79 | 114 (26.1%) | 21 (30.9%) | 3.547 (1.292-9.739) | 0.014 |
| ≥80 | 67 (15.3%) | 16 (23.5%) | 6.398 (1.973-20.740) | 0.002 |
| Admission GCS |  |  |  |  |
| 13-15 | 294 (67.3%) | 16 (23.5%) | Reference |  |
| 9-12 | 51 (11.7%) | 7 (10.3%) | 2.952 (1.065-8.181) | 0.037 |
| 3-8 | 92 (21.1%) | 45 (66.2%) | 18.944 (8.724-41.137) | <0.001 |
| Hypertension | 142 (32.5%) | 21 (30.9%) | 0.728 (0.356-1.487) | 0.384 |
| Atrial fibrillation | 55 (12.6%) | 15 (22.1%) | 1.596 (0.664-3.832) | 0.296 |
| Coronary heart disease | 49 (11.2%) | 14 (20.6%) | 2.219 (0.931-5.289) | 0.072 |
| Alcohol abuse | 122 (27.9%) | 26 (38.2%) | 1.861 (0.897-3.862) | 0.095 |
| Coagulopathy | 167 (38.2%) | 39 (57.4%) | 1.800 (0.875-3.701) | 0.110 |
| Coagulopathy correction | 152 (34.8%) | 30 (44.1%) | 0.545 (0.271-1.094) | 0.088 |
| Ventriculostomy | 11 (2.5%) | 3 (4.4%) | 1.967 (0.460-8.418) | 0.362 |
| Hemorrhage volume (ml), mean (95% CI) | 111.9 (102.0-121.8) | 142.0 (113.2-170.7) | NA^a^ | NA^a^ |
| Hemorrhage volume (ml) |  |  |  |  |
| 0-50 | 201 (46.0%) | 19 (27.9%) | Reference |  |
| 51-100 | 55 (12.6%) | 13 (19.1%) | 1.566 (0.634-3.868) | 0.331 |
| 101-200 | 109 (24.9%) | 18 (26.5%) | 1.148 (0.512-2.577) | 0.737 |
| >200 | 72 (16.5%) | 18 (26.5%) | 1.307 (0.560-3.049) | 0.536 |

OR = odds ratio, p = p-value, CI = confidence interval, GCS = Glasgow Coma Scale, NA^a^ = not included in the regression model due to categorized parameter of the same value
