# Supplementary material for: Coagulopathy and its effect on treatment and mortality in patients with traumatic intracranial hemorrhage
Source: Acta Neurochir (Wien). 2021 Mar 23;163(5):1391–401. doi: 10.1007/s00701-021-04808-0 (PMC8053656; doi:10.1007/s00701-021-04808-0)
Supplement: Supplementary file 4 — (DOCX 13 kb) [file 701_2021_4808_MOESM4_ESM.docx]

**Online Resource 4.** **Table.**

Multivariable analysis of factors associated with 30-day mortality in the entire study cohort (n=505). Sensitivity analysis with neurosurgical hematoma evacuation, without coagulopathy correction. Odds ratios from a logistic regression model: adjusted for all the given variables.

| **Variable** | **Alive**  **N=437 (86.5%)** | **Dead**  **N=68 (13.5%)** | **Multivariable OR (95% CI)** | **Multivariable p** |
| --- | --- | --- | --- | --- |
| Male gender | 282 (64.5%) | 49 (72.1%) | 1.528 (0.742-3.145) | 0.250 |
| Age, mean (95% CI) | 62.3 (60.4-64.3) | 63.5 (58.8-68.2) | NA^a^ | NA^a^ |
| Age group |  |  |  |  |
| <50 | 128 (29.3%) | 12 (17.6%) | Reference |  |
| 50-64 | 128 (29.3%) | 19 (27.9%) | 1.452 (0.569-3.703) | 0.435 |
| 65-79 | 114 (26.1%) | 21 (30.9%) | 3.421 (1.207-9.696) | 0.021 |
| ≥80 | 67 (15.3%) | 16 (23.5%) | 6.278 (1.878-20.992) | 0.003 |
| Admission GCS |  |  |  |  |
| 13-15 | 294 (67.3%) | 16 (23.5%) | Reference |  |
| 9-12 | 51 (11.7%) | 7 (10.3%) | 2.451 (0.853-7.046) | 0.096 |
| 3-8 | 92 (21.1%) | 45 (66.2%) | 13.861 (6.493-29.590) | <0.001 |
| Hypertension | 142 (32.5%) | 21 (30.9%) | 0.778 (0.364-1.663) | 0.517 |
| Atrial fibrillation | 55 (12.6%) | 15 (22.1%) | 1.460 (0.590-3.615) | 0.413 |
| Coronary heart disease | 49 (11.2%) | 14 (20.6%) | 1.995 (0.788-5.050) | 0.145 |
| Alcohol abuse | 122 (27.9%) | 26 (38.2%) | 1.976 (0.923-4.232) | 0.080 |
| Coagulopathy | 167 (38.2%) | 39 (57.4%) | 1.445 (0.689-3.028) | 0.330 |
| Hematoma evacuation | 248 (56.8%) | 24 (35.3%) | 0.130 (0.057-0.294) | <0.001 |
| Ventriculostomy | 11 (2.5%) | 3 (4.4%) | 2.693 (0.607-11.939) | 0.192 |
| Hemorrhage volume (ml), mean (95% CI) | 111.9 (102.0-121.8) | 142.0 (113.2-170.7) | NA^a^ | NA^a^ |
| Hemorrhage volume (ml) |  |  |  |  |
| 0-50 | 201 (46.0%) | 19 (27.9%) | Reference |  |
| 51-100 | 55 (12.6%) | 13 (19.1%) | 2.789 (1.071-7.260) | 0.036 |
| 101-200 | 109 (24.9%) | 18 (26.5%) | 3.920 (1.454-10.566) | 0.007 |
| >200 | 72 (16.5%) | 18 (26.5%) | 4.718 (1.656-13.445) | 0.004 |

OR = odds ratio, p = p-value, CI = confidence interval, GCS = Glasgow Coma Scale, NA^a^ = not included in the regression model due to categorized parameter of the same value
