# Supplementary material for: Coagulopathy and its effect on treatment and mortality in patients with traumatic intracranial hemorrhage
Source: Acta Neurochir (Wien). 2021 Mar 23;163(5):1391–401. doi: 10.1007/s00701-021-04808-0 (PMC8053656; doi:10.1007/s00701-021-04808-0)
Supplement: Supplementary file 5 — (DOCX 12 kb) [file 701_2021_4808_MOESM5_ESM.docx]

**Online Resource 5. Table.**

Univariable analysis of factors associated with 30-day mortality in the entire study cohort (n=505) divided into coagulopathy subgroups with operative descriptive values. Odds ratios from a logistic regression model: analysing each variable separately.

| **Coagulopathy groups** | **Neurosurgical operation (hematoma evacuation) N=272 (53.9%)** | **No operation N=233 (46.1%)** | **Alive**  **N=437 (86.5%)** | **Dead**  **N=68 (13.5%)** | **Univariable OR (95% CI)** | **Univariable p** |
| --- | --- | --- | --- | --- | --- | --- |
| No coagulopathy (N=299) | 148 (54.4%) | 151 (64.8%) | 270 (61.8%) | 29 (42.6%) | Reference |  |
| Medication-induced (N=81) | 44 (16.2%) | 37 (15.9%) | 67 (15.3%) | 14 (20.6%) | 1.945 (0.974-3.885) | 0.059 |
| Spontaneous (N=45) | 28 (10.3%) | 17  (7.3%) | 8 (11.8%) | 37 (8.5%) | 2.013 (0.856-4.733) | 0.109 |
| Both (N=80) | 52 (19.1%) | 28 (12.0%) | 17 (25.0%) | 63 (14.4%) | 2.512 (1.300-4.854) | 0.006 |

OR = odds ratio, p = p-value, CI = confidence interval
