# Supplementary material for: Coagulopathy and its effect on treatment and mortality in patients with traumatic intracranial hemorrhage
Source: Acta Neurochir (Wien). 2021 Mar 23;163(5):1391–401. doi: 10.1007/s00701-021-04808-0 (PMC8053656; doi:10.1007/s00701-021-04808-0)
Supplement: Supplementary file 6 — (DOCX 14 kb) [file 701_2021_4808_MOESM6_ESM.docx]

**Online Resource 6. Table.**

Multivariable analysis of factors associated with 30-day mortality in the entire study cohort (n=505) (coagulopathy subgroups included). Odds ratios from a logistic regression model: adjusted for all the given variables.

| **Variable** | **Alive**  **N=437 (86.5%)** | **Dead**  **N=68 (13.5%)** | **Multivariable OR (95% CI)** | **Multivariable p** |
| --- | --- | --- | --- | --- |
| Male gender | 282 (64.5%) | 49 (72.1%) | 1.538 (0.744-3.183) | 0.245 |
| Age, mean (95% CI) | 62.3 (60.4-64.3) | 63.5 (58.8-68.2) | NA^a^ | NA^a^ |
| Age group |  |  |  |  |
| <50 | 128 (29.3%) | 12 (17.6%) | Reference |  |
| 50-64 | 128 (29.3%) | 19 (27.9%) | 1.463 (0.567-3.771) | 0.431 |
| 65-79 | 114 (26.1%) | 21 (30.9%) | 3.536 (1.228-10.180) | 0.019 |
| ≥80 | 67 (15.3%) | 16 (23.5%) | 6.086 (1.782-20.791) | 0.004 |
| Admission GCS |  |  |  |  |
| 13-15 | 294 (67.3%) | 16 (23.5%) | Reference |  |
| 9-12 | 51 (11.7%) | 7 (10.3%) | 2.658 (0.904-7.819) | 0.076 |
| 3-8 | 92 (21.1%) | 45 (66.2%) | 15.400 (6.903-34.358) | <0.001 |
| Hypertension | 142 (32.5%) | 21 (30.9%) | 0.817 (0.378-1.769) | 0.609 |
| Atrial fibrillation | 55 (12.6%) | 15 (22.1%) | 1.820 (0.558-5.940) | 0.321 |
| Coronary heart disease | 49 (11.2%) | 14 (20.6%) | 2.094 (0.812-5.403) | 0.126 |
| Alcohol abuse | 122 (27.9%) | 26 (38.2%) | 1.924 (0.888-4.172) | 0.097 |
| Coagulopathy group |  |  |  |  |
| No coagulopathy | 270 (61.8%) | 29 (42.6%) | Reference |  |
| Medication-induced | 67 (15.3%) | 14 (20.6%) | 1.415 (0.529-3.784) | 0.489 |
| Spontaneous | 8 (11.8%) | 37 (8.5%) | 1.854 (0.650-5.286) | 0.248 |
| Both | 17 (25.0%) | 63 (14.4%) | 1.355 (0.353-5.200) | 0.657 |
| Coagulopathy correction | 152 (34.8%) | 30 (44.1%) | 0.711 (0.316-1.600) | 0.410 |
| Hematoma evacuation | 248 (56.8%) | 24 (35.3%) | 0.135 (0.059-0.311) | <0.001 |
| Ventriculostomy | 11 (2.5%) | 3 (4.4%) | 2.886 (0.630-13.216) | 0.172 |
| Hemorrhage volume (ml), mean (95% CI) | 111.9 (102.0-121.8) | 142.0 (113.2-170.7) | NA^a^ | NA^a^ |
| Hemorrhage volume (ml) |  |  |  |  |
| 0-50 | 201 (46.0%) | 19 (27.9%) | Reference |  |
| 51-100 | 55 (12.6%) | 13 (19.1%) | 2.681 (1.021-7.041) | 0.045 |
| 101-200 | 109 (24.9%) | 18 (26.5%) | 3.875 (1.434-10.471) | 0.008 |
| >200 | 72 (16.5%) | 18 (26.5%) | 4.578 (1.578-13.280) | 0.005 |

OR = odds ratio, p = p-value, CI = confidence interval, GCS = Glasgow Coma Scale, NA^a^ = not included in the regression model due to categorized parameter of the same value
