# Supplementary material for: Coagulopathy and its effect on treatment and mortality in patients with traumatic intracranial hemorrhage
Source: Acta Neurochir (Wien). 2021 Mar 23;163(5):1391–401. doi: 10.1007/s00701-021-04808-0 (PMC8053656; doi:10.1007/s00701-021-04808-0)
Supplement: Supplementary file 7 — (DOCX 14 kb) [file 701_2021_4808_MOESM7_ESM.docx]

**Online Resource 7. Table.**

Multivariable analysis of factors associated with 30-day mortality in the entire study cohort (n=505) (coagulopathy subgroups included). Sensitivity analysis without coagulopathy correction and neurosurgical hematoma evacuation. Odds ratios from a logistic regression model: adjusted for all the given variables.

| **Variable** | **Alive**  **N=437 (86.5%)** | **Dead**  **N=68 (13.5%)** | **Multivariable OR (95% CI)** | **Multivariable p** |
| --- | --- | --- | --- | --- |
| Male gender | 282 (64.5%) | 49 (72.1%) | 1.374 (0.695-2.717) | 0.361 |
| Age, mean (95% CI) | 62.3 (60.4-64.3) | 63.5 (58.8-68.2) | NA^a^ | NA^a^ |
| Age group |  |  |  |  |
| <50 | 128 (29.3%) | 12 (17.6%) | Reference |  |
| 50-64 | 128 (29.3%) | 19 (27.9%) | 1.585 (0.638-3.937) | 0.321 |
| 65-79 | 114 (26.1%) | 21 (30.9%) | 3.548 (1.291-9.755) | 0.014 |
| ≥80 | 67 (15.3%) | 16 (23.5%) | 6.978 (2.157-22.573) | 0.001 |
| Admission GCS |  |  |  |  |
| 13-15 | 294 (67.3%) | 16 (23.5%) | Reference |  |
| 9-12 | 51 (11.7%) | 7 (10.3%) | 2.657 (0.966-7.304) | 0.058 |
| 3-8 | 92 (21.1%) | 45 (66.2%) | 16.105 (7.660-33.860) | <0.001 |
| Hypertension | 142 (32.5%) | 21 (30.9%) | 0.681 (0.333-1.394) | 0.293 |
| Atrial fibrillation | 55 (12.6%) | 15 (22.1%) | 1.274 (0.443-3.666) | 0.653 |
| Coronary heart disease | 49 (11.2%) | 14 (20.6%) | 2.171 (0.899-5.245) | 0.085 |
| Alcohol abuse | 122 (27.9%) | 26 (38.2%) | 1.914 (0.918-3.987) | 0.083 |
| Coagulopathy group |  |  |  |  |
| No coagulopathy | 270 (61.8%) | 29 (42.6%) | Reference |  |
| Medication-induced | 67 (15.3%) | 14 (20.6%) | 1.780 (0.717-4.419) | 0.214 |
| Spontaneous | 8 (11.8%) | 37 (8.5%) | 1.298 (0.488-3.450) | 0.602 |
| Both | 17 (25.0%) | 63 (14.4%) | 1.596 (0.516-4.939) | 0.417 |
| Ventriculostomy | 11 (2.5%) | 3 (4.4%) | 1.684 (0.399-7.099) | 0.478 |
| Hemorrhage volume (ml), mean (95% CI) | 111.9 (102.0-121.8) | 142.0 (113.2-170.7) | NA^a^ | NA^a^ |
| Hemorrhage volume (ml) |  |  |  |  |
| 0-50 | 201 (46.0%) | 19 (27.9%) | Reference |  |
| 51-100 | 55 (12.6%) | 13 (19.1%) | 1.628 (0.663-3.999) | 0.288 |
| 101-200 | 109 (24.9%) | 18 (26.5%) | 1.100 (0.491-2.463) | 0.817 |
| >200 | 72 (16.5%) | 18 (26.5%) | 1.331 (0.570-3.104) | 0.508 |

OR = odds ratio, p = p-value, CI = confidence interval, GCS = Glasgow Coma Scale, NA^a^ = not included in the regression model due to categorized parameter of the same value
