# Supplementary material for: Coagulopathy and its effect on treatment and mortality in patients with traumatic intracranial hemorrhage
Source: Acta Neurochir (Wien). 2021 Mar 23;163(5):1391–401. doi: 10.1007/s00701-021-04808-0 (PMC8053656; doi:10.1007/s00701-021-04808-0)
Supplement: Supplementary file 8 — (DOCX 14 kb) [file 701_2021_4808_MOESM8_ESM.docx]

**Online Resource 8. Table.**

Multivariable analysis of factors associated with 30-day mortality in the entire study cohort (n=505) (coagulopathy subgroups included). Sensitivity analysis with coagulopathy correction, without neurosurgical hematoma evacuation. Odds ratios from a logistic regression model: adjusted for all the given variables.

| **Variable** | **Alive**  **N=437 (86.5%)** | **Dead**  **N=68 (13.5%)** | **Multivariable OR (95% CI)** | **Multivariable p** |
| --- | --- | --- | --- | --- |
| Male gender | 282 (64.5%) | 49 (72.1%) | 1.437 (0.720-2.870) | 0.304 |
| Age, mean (95% CI) | 62.3 (60.4-64.3) | 63.5 (58.8-68.2) | NA^a^ | NA^a^ |
| Age group |  |  |  |  |
| <50 | 128 (29.3%) | 12 (17.6%) | Reference |  |
| 50-64 | 128 (29.3%) | 19 (27.9%) | 1.568 (0.625-3.937) | 0.338 |
| 65-79 | 114 (26.1%) | 21 (30.9%) | 3.489 (1.255-9.704) | 0.017 |
| ≥80 | 67 (15.3%) | 16 (23.5%) | 6.332 (1.936-20.708) | 0.002 |
| Admission GCS |  |  |  |  |
| 13-15 | 294 (67.3%) | 16 (23.5%) | Reference |  |
| 9-12 | 51 (11.7%) | 7 (10.3%) | 2.878 (1.032-8.023) | 0.043 |
| 3-8 | 92 (21.1%) | 45 (66.2%) | 18.871 (8.716-40.857) | <0.001 |
| Hypertension | 142 (32.5%) | 21 (30.9%) | 0.720 (0.349-1.484) | 0.373 |
| Atrial fibrillation | 55 (12.6%) | 15 (22.1%) | 1.369 (0.466-4.022) | 0.568 |
| Coronary heart disease | 49 (11.2%) | 14 (20.6%) | 2.194 (0.908-5.302) | 0.081 |
| Alcohol abuse | 122 (27.9%) | 26 (38.2%) | 1.910 (0.913-3.996) | 0.086 |
| Coagulopathy group |  |  |  |  |
| No coagulopathy | 270 (61.8%) | 29 (42.6%) | Reference |  |
| Medication-induced | 67 (15.3%) | 14 (20.6%) | 1.756 (0.709-4.353) | 0.224 |
| Spontaneous | 8 (11.8%) | 37 (8.5%) | 1.694 (0.597-4.803) | 0.322 |
| Both | 17 (25.0%) | 63 (14.4%) | 2.293 (0.673-7.812) | 0.185 |
| Coagulopathy correction | 152 (34.8%) | 30 (44.1%) | 0.521 (0.246-1.107) | 0.090 |
| Ventriculostomy | 11 (2.5%) | 3 (4.4%) | 2.027 (0.466-8.817) | 0.346 |
| Hemorrhage volume (ml), mean (95% CI) | 111.9 (102.0-121.8) | 142.0 (113.2-170.7) | NA^a^ | NA^a^ |
| Hemorrhage volume (ml) |  |  |  |  |
| 0-50 | 201 (46.0%) | 19 (27.9%) | Reference |  |
| 51-100 | 55 (12.6%) | 13 (19.1%) | 1.546 (0.624-3.831) | 0.347 |
| 101-200 | 109 (24.9%) | 18 (26.5%) | 1.140 (0.508-2.562) | 0.750 |
| >200 | 72 (16.5%) | 18 (26.5%) | 1.283 (0.548-3.001) | 0.566 |

OR = odds ratio, p = p-value, CI = confidence interval, GCS = Glasgow Coma Scale, NA^a^ = not included in the regression model due to categorized parameter of the same value
