# Supplementary material for: Coagulopathy and its effect on treatment and mortality in patients with traumatic intracranial hemorrhage
Source: Acta Neurochir (Wien). 2021 Mar 23;163(5):1391–401. doi: 10.1007/s00701-021-04808-0 (PMC8053656; doi:10.1007/s00701-021-04808-0)
Supplement: Supplementary file 9 — (DOCX 14 kb) [file 701_2021_4808_MOESM9_ESM.docx]

**Online Resource 9. Table.**

Multivariable analysis of factors associated with 30-day mortality in the entire study cohort (n=505) (coagulopathy subgroups included). Sensitivity analysis with neurosurgical hematoma evacuation, without coagulopathy correction. Odds ratios from a logistic regression model: adjusted for all the given variables.

| **Variable** | **Alive**  **N=437 (86.5%)** | **Dead**  **N=68 (13.5%)** | **Multivariable OR (95% CI)** | **Multivariable p** |
| --- | --- | --- | --- | --- |
| Male gender | 282 (64.5%) | 49 (72.1%) | 1.519 (0.737-3.132) | 0.257 |
| Age, mean (95% CI) | 62.3 (60.4-64.3) | 63.5 (58.8-68.2) | NA^a^ | NA^a^ |
| Age group |  |  |  |  |
| <50 | 128 (29.3%) | 12 (17.6%) | Reference |  |
| 50-64 | 128 (29.3%) | 19 (27.9%) | 1.454 (0.568-3.720) | 0.435 |
| 65-79 | 114 (26.1%) | 21 (30.9%) | 3.512 (1.223-10.086) | 0.020 |
| ≥80 | 67 (15.3%) | 16 (23.5%) | 6.386 (1.883-21.657) | 0.003 |
| Admission GCS |  |  |  |  |
| 13-15 | 294 (67.3%) | 16 (23.5%) | Reference |  |
| 9-12 | 51 (11.7%) | 7 (10.3%) | 2.520 (0.866-7.331) | 0.090 |
| 3-8 | 92 (21.1%) | 45 (66.2%) | 14.011 (6.511-30.150) | <0.001 |
| Hypertension | 142 (32.5%) | 21 (30.9%) | 0.794 (0.368-1.713) | 0.557 |
| Atrial fibrillation | 55 (12.6%) | 15 (22.1%) | 1.721 (0.539-5.497) | 0.359 |
| Coronary heart disease | 49 (11.2%) | 14 (20.6%) | 2.064 (0.799-5.329) | 0.134 |
| Alcohol abuse | 122 (27.9%) | 26 (38.2%) | 1.918 (0.885-4.159) | 0.099 |
| Coagulopathy group |  |  |  |  |
| No coagulopathy | 270 (61.8%) | 29 (42.6%) | Reference |  |
| Medication-induced | 67 (15.3%) | 14 (20.6%) | 1.415 (0.528-3.793) | 0.491 |
| Spontaneous | 8 (11.8%) | 37 (8.5%) | 1.622 (0.603-4.362) | 0.338 |
| Both | 17 (25.0%) | 63 (14.4%) | 1.133 (0.321-4.003) | 0.846 |
| Hematoma evacuation | 248 (56.8%) | 24 (35.3%) | 0.127 (0.056-0.290) | <0.001 |
| Ventriculostomy | 11 (2.5%) | 3 (4.4%) | 2.669 (0.598-11.917) | 0.198 |
| Hemorrhage volume (ml), mean (95% CI) | 111.9 (102.0-121.8) | 142.0 (113.2-170.7) | NA^a^ | NA^a^ |
| Hemorrhage volume (ml) |  |  |  |  |
| 0-50 | 201 (46.0%) | 19 (27.9%) | Reference |  |
| 51-100 | 55 (12.6%) | 13 (19.1%) | 2.811 (1.079-7.322) | 0.034 |
| 101-200 | 109 (24.9%) | 18 (26.5%) | 4.005 (1.481-10.830) | 0.006 |
| >200 | 72 (16.5%) | 18 (26.5%) | 4.871 (1.692-14.019) | 0.003 |

OR = odds ratio, p = p-value, CI = confidence interval, GCS = Glasgow Coma Scale, NA^a^ = not included in the regression model due to categorized parameter of the same value
