# Supplementary material for: Coagulopathy and its effect on treatment and mortality in patients with traumatic intracranial hemorrhage
Source: Acta Neurochir (Wien). 2021 Mar 23;163(5):1391–401. doi: 10.1007/s00701-021-04808-0 (PMC8053656; doi:10.1007/s00701-021-04808-0)
Supplement: Supplementary file 10 — (DOCX 12 kb) [file 701_2021_4808_MOESM10_ESM.docx]

**Online Resource 10. Table.**

Univariable analysis of factors associated with 30-day mortality in the entire study cohort (n=505) divided into alcohol abuse subgroups. Odds ratios from a logistic regression model: analysing each variable separately.

| **Alcohol abuse groups** | **Alive**  **N=437 (86.5%)** | **Dead**  **N=68 (13.5%)** | **Univariable OR (95% CI)** | **Univariable p** |
| --- | --- | --- | --- | --- |
| No alcohol abuse (N=357) | 315 (72.1%) | 42 (61.8%) | Reference |  |
| History (N=127) | 107 (24.5%) | 20 (29.4%) | 1.402 (0.788-2.493) | 0.250 |
| History and laboratory (N=12) | 8 (1.8%) | 4 (5.9%) | 3.750 (1.082-12.993) | 0.037 |
| History and laboratory and admission (N=9) | 7 (1.6%) | 2 (2.9%) | 2.143 (0.431-10.657) | 0.352 |

OR = odds ratio, p = p-value, CI = confidence interval, History = history of previous heavy alcohol consumption, Laboratory = admission laboratory values indication coagulopathy, Admission = over 1 per mille of alcohol in blood or breath
