# Supplementary material for: Coagulopathy and its effect on treatment and mortality in patients with traumatic intracranial hemorrhage
Source: Acta Neurochir (Wien). 2021 Mar 23;163(5):1391–401. doi: 10.1007/s00701-021-04808-0 (PMC8053656; doi:10.1007/s00701-021-04808-0)
Supplement: Supplementary file 11 — (DOCX 14 kb) [file 701_2021_4808_MOESM11_ESM.docx]

**Online Resource 11. Table.**

Multivariable analysis of factors associated with 30-day mortality in the entire study cohort (n=505) (alcohol abuse subgroups included). Odds ratios from a logistic regression model: adjusted for all the given variables.

| **Variable** | **Alive**  **N=437 (86.5%)** | **Dead**  **N=68 (13.5%)** | **Multivariable OR (95% CI)** | **Multivariable p** |
| --- | --- | --- | --- | --- |
| Male gender | 282 (64.5%) | 49 (72.1%) | 1.512 (0.729-3.137) | 0.266 |
| Age, mean (95% CI) | 62.3 (60.4-64.3) | 63.5 (58.8-68.2) | NA^a^ | NA^a^ |
| Age group |  |  |  |  |
| <50 | 128 (29.3%) | 12 (17.6%) | Reference |  |
| 50-64 | 128 (29.3%) | 19 (27.9%) | 1.478 (0.573-3.810) | 0.419 |
| 65-79 | 114 (26.1%) | 21 (30.9%) | 3.500 (1.226-9.987) | 0.019 |
| ≥80 | 67 (15.3%) | 16 (23.5%) | 6.094 (1.794-20.699) | 0.004 |
| Admission GCS |  |  |  |  |
| 13-15 | 294 (67.3%) | 16 (23.5%) | Reference |  |
| 9-12 | 51 (11.7%) | 7 (10.3%) | 2.591 (0.884-7.592) | 0.083 |
| 3-8 | 92 (21.1%) | 45 (66.2%) | 15.362 (6.919-34.106) | <0.001 |
| Hypertension | 142 (32.5%) | 21 (30.9%) | 0.798 (0.372-1.714) | 0.563 |
| Atrial fibrillation | 55 (12.6%) | 15 (22.1%) | 1.615 (0.620-4.210) | 0.326 |
| Coronary heart disease | 49 (11.2%) | 14 (20.6%) | 2.057 (0.811-5.216) | 0.129 |
| Alcohol abuse groups |  |  |  |  |
| No alcohol abuse | 315 (72.1%) | 42 (61.8%) | Reference |  |
| History | 107 (24.5%) | 20 (29.4%) | 1.828 (0.824-4.053) | 0.138 |
| History and laboratory | 8 (1.8%) | 4 (5.9%) | 2.820 (0.511-15.573) | 0.234 |
| History and laboratory and admission | 7 (1.6%) | 2 (2.9%) | 3.423 (0.341-34.397) | 0.296 |
| Coagulopathy | 167 (38.2%) | 39 (57.4%) | 1.465 (0.658-3.262) | 0.349 |
| Coagulopathy correction | 152 (34.8%) | 30 (44.1%) | 0.731 (0.343-1.558) | 0.417 |
| Hematoma evacuation | 248 (56.8%) | 24 (35.3%) | 0.136 (0.060-0.312) | <0.001 |
| Ventriculostomy | 11 (2.5%) | 3 (4.4%) | 2.861 (0.635-12.894) | 0.171 |
| Hemorrhage volume (ml), mean (95% CI) | 111.9 (102.0-121.8) | 142.0 (113.2-170.7) | NA^a^ | NA^a^ |
| Hemorrhage volume (ml) |  |  |  |  |
| 0-50 | 201 (46.0%) | 19 (27.9%) | Reference |  |
| 51-100 | 55 (12.6%) | 13 (19.1%) | 2.851 (1.085-7.492) | 0.034 |
| 101-200 | 109 (24.9%) | 18 (26.5%) | 4.165 (1.566-11.077) | 0.004 |
| >200 | 72 (16.5%) | 18 (26.5%) | 5.393 (1.886-15.419) | 0.002 |

OR = odds ratio, p = p-value, CI = confidence interval, GCS = Glasgow Coma Scale, NA^a^ = not included in the regression model due to categorized parameter of the same value, History = history of previous heavy alcohol consumption, Laboratory = admission laboratory values indication coagulopathy, Admission = over 1 per mille of alcohol in blood or breath
